# Susceptibility of processed and stored cassava, plantain, yam, and cocoyam to coffee bean weevil (*Araecerus fasciculatus* De Geer)

**DOI:** 10.1186/s41936-023-00341-x

**Published:** 2023-06-01

**Authors:** Joy Oluwafunke Adeoti, Olaniyi Charles Ogungbite, Olufemi Samson Salami, Olusola Olasumbo Odeyemi

**Affiliations:** Department of Biology, Federal University of Technology, Akure, Nigeria

**Keywords:** *Araecerus fasciculatus*, Coffee bean weevil, Tannin, Alkaloid, Phytate, Oxalate, Yam, Cassava, Cocoyam, Plantain

## Abstract

**Background:**

Coffee bean weevil (*Araecerus fasciculatus* DeGeer) (Coleoptera: Anthribidae) infestation is a major challenge affecting processed, dried, and stored foods globally. However, the growth performance of this insect pest in processed and stored cassava, plantain, yam, and cocoyam is poorly understood. Resolving this will guide future biotechnological efforts on these food products. In the study, the susceptibility rate of the processed and stored product of cassava, yam, cocoyam, and plantain to the growth performance of the coffee bean weevil at laboratory conditions (20 ± 5 °C and 70 ± 5% R.H.) was investigated. The anti-nutritional (phytate, alkaloids, saponin, tannin, and oxalate) contents of this stored food were used to characterize the survival rate of *A. fasciculatus.* The study was carried out for 6 months between December 2012 and May 2013.

**Results:**

Results show that the adult longevity, growth, and feeding of the adult *A. fasciculatus* decrease in order from Cassava, Plantain, Yam to Cocoyam. Cocoyam has the highest tannin, alkaloid, and phytate values, which are possible factors that inhibited the growth of the larvae, pupae, and adults’ *A. fasciculatus*. The highest weight loss occurred in cassava followed by plantain. The anti-nutritional factors determine the hatchability of the *A. fasciculatus* eggs and throughout its development stages. This study revealed that processed and stored products (e.g., cocoyam) with high anti-nutritional factors can survive *A. fasciculatus* under prolonged storage.

**Conclusions:**

From this study, it is observed that high anti-nutritional compounds in the stored food products inhibit the growth of *Araecerus fasciculatus*, especially in cocoyam. The result is important in food security and management in developing countries where food insecurity has become a major challenge.

## Background

Apart from the changing global climate, war, and conflicts, where the former has contributed to the spread of insect pest distribution and the latter disrupting supply chain, pest infestation is one of the major challenges affecting processed and stored food products (Lin et al., 2022; Skendžić et al., 2021). Climate change has direct impact on the biological activities of many stored products insect pest. Shrestha (2019) reported that climate change increases both the population and damage potential of insect pest (Dubey et al., 2008; Rajashekar & Shivanandappa, 2010). The attack on most stored food products has led to serious food damages with significant impacts on global food security (FAO, 2006) because most storage pests can increase in numbers drastically within a relatively short time (Poswal et al., 1993). This challenge is more significant in many developing countries where there are no good and improved post-harvest management systems for their processed and stored products (Alabi et al., 2006).

Common insect pests that attack dried stored foods include beetles, moths, and mites have been widely studied. Some of these studies (e.g., Emeasor et al., 2005; Odeyemi et al., 2006, 2013) have shown that these pests feed on the endosperm causing loss of weight and quality, while others feed on the germ, resulting in poor seed germination and less viability. While many of these insect pests have received extensive research attention (e.g., Ashamo & Odeyemi, 2001; Emeasor et al., 2005; Odeyemi et al., 2006; Akinneye & Ogungbite, 2013), only *A. fasciculatus* growth performance on processed root crops is not well understood (Sayed, 1935). Augustine et al. (2016) reported that processing of food increases the shelf life and nutritional value of the processed food.

Significant damage of *A. fasciculatus* on stored dried products has been reported (Chijindu & Boateng, 2008), but it is not clear how *A. fasciculatus* survives during its activities with stored food products.

Addressing this challenge involves understanding the growth performance of *A. fasciculatus* under food storage conditions and the susceptibility of this stored food to *A. fasciculatus* attack. We selected four major starchy staple stored processed foods comprising cassava (*Manihot esculenta Crantz*), white yam (*Dioscorea rotundata* L*.)*, plantain (*Musa paradisiaca* L*.*), and cocoyam (*Colocasia esculenta* L.) and quantitatively measure the growth performance of *A. fasciculatus* on the dried cassava, yam, cocoyam, and plantain. These are important and common staple foods which are widely consumed in Sub-Saharan Africa and available almost all year round in different seasons which price have been greatly affected by the impact of the COVID-19 pandemic (Okou et al., 2022). We focused on how food anti-nutritional factors affect *A. fasciculatus* growth. The outcome of this study is important for post-harvest storage management and for food security in countries where post-harvest management remains a challenge.

## Methods

### Preparation of food media

Four stored food products such as cassava, cocoyam, plantain, and yam chips were used for this study and were carried out in 6 months between December 2012 and May 2013. Each food medium was sized into 2-cm cubes using a Dexteria tube sizer (Dexteria corp., Japan) to avoid surface area bias. The chips were kept under freezing conditions at − 2 °C for 72 h in the refrigerator to disinfest each of the food samples from insect contaminants. The samples were removed and kept under ambient laboratory conditions (26 ± 2 °C, 70 ± 5% R.H.) until required for use. The moisture content of the food samples was determined using a moisture meter, and proximate analysis was carried out on them before infestation following AOAC (1990) methods.

### Preparation of insect culture

Sample of dried cassava chips (500 mg) infested with *A. fasciculatus* was acquired, and the adults of *A. fasciculatus* were cultured in the disinfested cassava, yam, cocoyam, and plantain chips under the ambient temperature of 28 ± 2 °C and relative humidity (RH) of 70 ± 5%; 250 g of each chip was sterilized in the refrigerator at − 2 °C for 72 h and brought to ambient temperature to thaw for 2 days. 50 g of each chip was weighed into hermetic containers covered with muslin cloth held tightly to cover the container from aeration. These containers were kept in an insect-rearing cage of dimension (55 cm × 60 cm × 90 cm) for 46 days to allow the growth of adult *A. fasciculatus*.

## Experimental procedure

### Adult longevity of coffee bean weevil on the different food media

10 g of each of the four different food chips (2 cm × 2 cm × 2 cm) was weighed into disposable plastic containers covered with muslin cloth and tightly held in place by rubber bands. Ten unsexed 0–24-h-old *A. fasciculatus* adults were introduced into the containers and allowed to feed for 10 days for acclimatization. The experiment was laid out in a completely randomized design and replicated three times. On the tenth day, the surviving and dead *A. fasciculatus* adults were counted and the percentages of adult survival were calculated.

### Developmental/susceptibility test experiment

50 g of each sun-dried food chip (2 cm × 2 cm × 2 cm) was weighed using electronic weighing balance into disposable 200-ml plastic containers. Newly emerged (0–24 h) adults of *A. fasciculatus* were examined under the binocular microscope and sorted out into different sexes as described in the previous literature (Halstead, 1986; Sayed, 1935). The sexes were confirmed by observing the abdominal features; the pygidium in the male is vertical and not distinctly visible dorsally, while the pygidium in females is inclined and distinctly visible dorsally.

Adult male and female sexes were introduced into each of the food media in a ratio of 2:3, respectively. The adults were carefully picked using a Carmel brush to avoid damage to any of its body parts. Muslin cloths were held in place to cover the containers. The experiment was carried out in three replicates. The containers were then arranged inside the insect-rearing cage in a complete randomized design. The whole experiment was left in the laboratory for 48 h before the adult insects introduced were removed after mating. The experimental setup was subsequently checked daily for adult emergence. During this experimental period, the number of eggs, larvae, pupae, and percentage of adult emergence were counted.

### Anti-nutritional properties of the food media

#### Determination of tannin

Finely powdered sample (2 g) of each sun-dried food product was weighed into a 50-ml sample bottle. According to Makker (2003), 10 ml of 70% aqueous acetone was added and covered. The bottle was put in an ice bath shaker and shaken for 2 h at 30 °C. Each solution was then centrifuged, and the supernatant was stored in ice. Subsequently, 0.2 ml of each solution was pipetted into the test tube and 0.8 ml of distilled water was added. Standard tannic acid solutions were prepared from 0.5 mg/ml of the stock, and the solution was made up to 1 ml with distilled water. 0.5 ml of Folin–Ciocalteu reagent was added to both sample and standard followed by 2.5 ml of 20% Na_2_CO_3_. The solutions were then vortexed and allowed to incubate for 40 min. at room temperature. The absorbance was read at 725 nm against a reagent blank concentration of the same solution from a standard tannic acid curve following the techniques of Makkar (2003).

#### Determination of phytate

Phytate was determined according to Wheeler and Ferrel (1971). 4 g of each food sample was soaked in 100 ml of 2% HCl for 3 h and then filtered through a No. 1 Whatman filter paper. After 25 ml was taken out of the filtrate and placed inside a conical flask, 5 ml of 0.3% of ammonium thiocyanate solution was added as an indicator. Also, 53.5 ml of distilled water was added to give it the proper acidity before it was titrated against 0.00566 g/ml of standard Iron (III) Chloride (FeCl_3_) solution that contains about 0.00195 g/ml of iron until a brownish-yellow coloration persists for 5 min (Wheeler & Ferrel, 1971).

#### Determination of oxalate

Oxalate was determined by soaking 1 g of each sample in 75 ml of 1.5N H_2_SO_4_ for 1 h and then filtered through a No. 1 Whatman filter paper. 25 ml was taken out of the filtrate and placed inside a conical flask, which was later titrated at about 80–90 °C against 0.1 m of KMnO_4_ until a pink coloration persisted for 15 secs as described by Day and Underwood (1986).

#### Determination of saponin

The spectrophotometric method of Brunner (1984) was used for Saponin determination. Two grams of the finely grounded sample was weighed into a 250-ml beaker, and then 100 ml of Isobutyl alcohol (But-2-ol) was later added. The mixture was later shaken for 5 min to ensure uniform mixing. The mixture was then filtered using No. 1 Whatman filter paper into a 100-ml beaker containing 20 ml of 40% saturated solution of magnesium carbonate (MgCO_3_). The mixture obtained again was filtered through No. 1 Whatman filter paper to obtain a clear colorless solution. 1 ml of the colorless solution was taken into a 50-ml volumetric flask using a pipette, and 2 ml of 5% iron (III) chloride (FeCl_3_) solution was added and made up to the mark with distilled water. It was allowed to stand for 30 min for the color to develop. The absorbance was read against the blank at 380 nm.

#### Determination of alkaloid

Following the method of Harborne (1973), each food sample weighing 5 g was added into a 250-ml beaker and 200 ml of 10% acetic acid and ethanol was added and allowed to stand for 4 min. This was later filtered, and the extract was concentrated on a water bath to one-quarter of the original volume. Concentrated ammonium hydroxide (NH_4_OH) was added dropwise to the extract until the precipitation was completed. The whole solution was allowed to settle, and the precipitate was collected and washed with dilute ammonium hydroxide and then filtered. The residue obtained (alkaloid) was then dried and weighed, and the percentage alkaloid was determined using the following equation:

1 $$\% \,\text{alkaloid}= \frac{{W}_{3}-{W}_{2}}{{W}_{1}}\times 100$$

### Proximate analysis

This was carried out using Wande experimentation AOAC (1990) methods. This analysis was carried out on the four-food media (cassava, yam, cocoyam, and plantain).

#### Determination of ash content

Clean dried crucibles were weighed (*W*_1_); 1 g of each of the samples was put into the crucible and weighed (*W*_2_). The crucibles were then heated in the muffle furnace set at 500 °C. Heating was continued until a light gray or white ash was obtained. The crucibles were then removed from the furnace, cooled in desiccators to room temperature, and weighed (*W*_3_). Cooling and weighing were continued until a constant weight was obtained. The percentage of ash content was determined using the following equation:

2 $$\% \,\text{Ash Content}= \frac{{W}_{3}-{W}_{1}}{{W}_{2 }-{W}_{1} }\times 100$$

#### Determination of crude fiber

1 g of the defatted samples of the four-food media was put inside a clean, dried, and well-labeled conical flask and weighed (*W*_1_). 200 ml of 1.25% H_2_SO_4_ was added to the samples in the conical flask and was boiled for 30 min. The solutions were filled (to remove the fat and sugar) and the residue was put back into the conical flask and distilled water was used to rinse them. 200 ml of 1.25% of NaOH solution was added in each sample and heated to boil for 30 min. The boiled samples were then filtered with ethanol and 10% HCL was added to rinse them. The residue of each sample was put into a crucible and placed into the oven for 3 h at 105 °C. The samples were then ashed in the muffle furnace 3 h at 500 °C. The samples were then removed, cooled in desiccators, and weighed (*W*_3_). The percentage of crude fiber was determined using the following equation:

3 $$\%\, \text{Crude Fibre}= \frac{{W}_{2}-{W}_{3}}{{W}_{2 } }\times 100$$

#### Determination of moisture content

Clean, well-labeled, and dry Petri dishes were oven-dried at selected drying temperature and weighed, and their respective weights were recorded (*W*_1_). 5 g of the four-food media was weighed into respective Petri dishes (*W*_2_), and it was transferred into desiccators immediately to avoid moisture absorption from the atmosphere. The Petri dishes were transferred to the oven at 105 °C and dried for 3 h. After drying, they were cooled in the desiccators and weighed; the processes of heating and cooling and weighing were continued until a constant weight was obtained (*W*_3_). The percentage of moisture content was determined using the following equation:

4 $$\%\, \text{Moisture Content}= \frac{{W}_{1}-{W}_{3}}{{W}_{2 }-{W}_{1} }\times 100$$

#### Determination of fat content

The fat was extracted with petroleum ether (40–60%) boiling range from dried residues obtained after the determination of the moisture content, the solvent was removed by evaporation, and the fat residue was weighed. The Soxhlet extraction method which was used could only give the approximate fat content in a sample. It is necessary to avoid the presence of water so that the water-soluble materials are not extracted along with the fat. Filter papers were weighed (*W*_1_), and 1 g of each of the samples was weighed into the filter papers, wrapped neatly with thread, and weighed (*W*_2_). The filter paper with the sample was inserted into the Soxhlet apparatus and extracted under reflux with petroleum ether boiling for 6 h. At the end of the extraction, the filter paper and their content were dried in the oven for 30 min at 100 °C to evaporate the solvents and weighed (*W*_3_). The percentage of fat content was determined using the following equation:

5 $$\%\, \text{Fat Content}= \frac{{W}_{2}-{W}_{3}}{{W}_{2 }-{W}_{1} }\times 100$$

#### Determination of protein content

This was determined using the Kjeldahl method. It involved three steps. The first stage is the digestion, 0.6 g of each of the four food samples was digested with a 10 ml of H_2_SO_4_ in a dry 500-ml Kjeldahl digestion flask tighter with a catalyst (Selenium). The mixture was then swirled together, and the flask was filtered with a loose pear stopper in an inclined position. It was then placed in a fume cupboard and heated gently gradually. The mixture was swirled and agitated; it was heated until a clear solution was obtained. The flask was allowed to cool after which the solution was diluted with tap water to 100 ml of which 10 ml was transferred into distillation Kjeldahl flask. The second stage was the distillation stage 40% NaOH solution was added to the cooled and diluted digested sample to make it alkaline. To the receiving flask, 25 ml of 2% Boric acid was added and few drops of screened methyl red indicator were also added to produce a pink coloration. The distillation was carried out with all joints tightened with the end of the delivery tube dipping below the boric acid solution. The third stage involves the titration in which NH3 receives in the acid solution as titrated with 0.1 M of HCL solution.

$$\begin{aligned} & \% \,{\text{Nitrogen}} = {\text{Volume}}\;{\text{of}}\;{\text{acid}}\;{\text{used }}* \, 0.00{14}*{1}00/{\text{weight of sample}} \\ & {1}\; {\text{ml}}\;{\text{of}}\; \, 0.{1}\; {\text{mHCL}} = 0.00{14}\;{\text{gN }}({\text{crude protein}} = \% {\text{ Nitrogen}}*{6}.{25}) \\ \end{aligned}$$

### Statistical analysis

Percentage data were arcsine-transformed before subjecting them to one-way ANOVA. The differences between means were compared using the new Duncan’s multiple range test (NDMRT) at a 5% level of significance. SPSS version 17 was used for the analysis.

## Results

### Mean survival rate and the development stages of *A. fasciculatus* in the different food media

The survival rate of 80% was recorded in cassava. Cocoyam and yam media recorded the least survival rate of 23.3%. Plantain recorded a survival rate of 36.7%, a value that is not significantly different from the survival rate (23.3%) in yam powder (Table 1). The fecundity rate of the insects is highest in cassava with a mean value of 4.7. Plantain and yam produced a fecundity values of 3.3 and 2.3, respectively: these values were not significantly different from 4.7 recorded in cassava.

**Table 1:** Mean survival rate of adult *A. fasciculatus* in four-food media

Food media	Survival rate	
	% Dead Insect	% Live Insect
Cassava	20.0 ± 0.58a	80.0 ± 0.58c
Cocoyam	76.7 ± 1.45c	23.3 ± 1.45a
Plantain	63.3 ± 0.33ab	36.7 ± 0.33b
Yam	66.7 ± 1.45bc	23.3 ± 0.88ab

Each data represents the mean ± 2*σ* of the three replicates. Mean followed by the same letter in the same column are not significantly different (*p* > 0.05) from each other by the new Duncan’s multiple range test

These results suggest that the mean survival rates in the same column (Table 1) are significantly different from each other (*p* < 0.05) except for those with the same letter, which are not significantly different. Therefore, the survival rates in cassava and the survival rates in plantain and yam are significantly different from each other, but not significantly different from each other within each group. Similarly, the survival rates in cocoyam are significantly different from those in the other three food media.

Overall, these results suggest that cassava may be a preferred food source making it more susceptible for adult *A. fasciculatus*, while cocoyam may be less suitable.

Cassava had the highest number of developmental stages, with the insect progressing through all four stages, while cocoyam had no developmental stages observed, indicating that it was not a suitable food source for *A. fasciculatus*. Plantain and yam had fewer developmental stages compared to cassava, with the insect reaching the adult stage after passing through three developmental stages. Insects that were developed in cassava had the highest number of laid eggs, larvae, pupae, and adults emerges of 4.7, 4.7, 4.0, and 3.7, respectively. (Table 2). However, insects that were developed in the cocoyam had the lowest number of all the life stages of the insects except the egg stage where it recorded 2.7 numbers of laid eggs and no other life stage. Cassava flour recorded the highest weight loss of 17.33%, while no weight loss was recorded in cocoyam. The weight loss was highest during the pupal stage in cassava, while it was highest during the larval stage in plantain. The weight loss is related to feeding activities of the larva and adult stages of the insect.

**Table 2:** Number of developmental stages of *A. fasciculatus* and their percentage weight loss

Food media	Developmental stages				Percentage weight loss
	Egg	Larvae	Pupae	Adult	
Cassava	4.7 ± 0.88a	4.7 ± 0.88b	4.0 ± 1.00b	3.7 ± 0.67b	17.3 ± 2.73c
Cocoyam	2.7 ± 0.33a	0.0 ± 0.00a	0.0 ± 0.00a	0.0 ± 0.00a	0.0 ± 0.00a
Plantain	3.3 ± 0.67a	2.3 ± 1.33ab	2.7 ± 1.33ab	0.67 ± 0.33a	7.0 ± 1.15b
Yam	2.3 ± 0.88a	1.7 ± 1.20ab	1.7 ± 1.20ab	0.3 ± 0.33a	0.7 ± 0.33a

Each data represents the mean ± 2*σ* of the three replicates. Mean followed by the same letter in the same column are not significantly different (*p* > 0.05) from each other by the new Duncan’s multiple range test

### Anti-nutritional compounds present in the four processed food media

The antinutrient properties varied significantly among the four-food media. Cocoyam had the highest tannin content of 11.2 mg/g while cassava recorded the lowest tannin content of 6.6 mg/g (Table 3). The oxalate content of the four-food media was relatively low; cocoyam recorded the lowest 1.4 oxalate content. Cassava recorded the lowest saponin content of 0.9 mg/g, while plantain had the highest levels of oxalate content of 14.5 mg/g. Yam had the lowest levels of all the antinutrients measured, except for phytate, which was highest in cocoyam recording 31.7 mg/g.

**Table 3:** Antinutrient properties of the four processed food media

Food media	Antinutrient (mg/g)				
	Tannin	Oxalate	Saponin	Alkaloid	Phytate
Cassava	6.6 ± 0.04a	1.7 ± 0.43a	0.9 ± 0.01a	0.0 ± 0.0a	14.8 ± 0.01b
Cocoyam	11.2 ± 0.17d	1.4 ± 0.03a	4.9 ± 0.03c	0.1 ± 0.06a	31.7 ± 0.26d
Plantain	7.7 ± 0.09b	1.7 ± 0.08a	14.5 ± 0.23d	0.0 ± 0.00a	15.7 ± 0.00c
Yam	10.9 ± 0.35c	1.9 ± 0.03a	4.3 ± 0.02b	0.0 ± 0.00a	13.18 ± 0.00a

Each data represents the mean ± 2*σ* of the three replicates. Mean followed by the same letter in the same column are not significantly different (*p* > 0.05) from each other by the new Duncan’s multiple range test

### Proximate analysis of the four-food media before *A. fasciculatus* infestation

Cassava had the highest amount of moisture content of 9.6% (Table 4). Yam recorded the lowest ash content of 1.7, and the lowest amount of fiber content (0.7) was observed in the plantain. Cocoyam recorded the lowest amount of fat content (4.1), and cassava had the lowest protein content of all the four-food media (2.9). The percentage of moisture and fat varied among the food media, but there were no significant differences between the means.

**Table 4:** Proximate analysis of the four processed food media before infestation of *A. fasciculatus*

Food media	Proximate				
	% Moisture	% Ash	%Fiber	%Fat	%Protein
Cassava	9.6 ± 0.41b	2.0 ± 0.11ab	1.8 ± 0.02c	4.3 ± 0.51a	2.9 ± 0.57c
Cocoyam	8.8 ± 0.23ab	1.8 ± 0.27a	1.5 ± 0.05b	4.1 ± 0.42a	7.0 ± 0.99a
Plantain	8.3 ± 0.36a	2.6 ± 0.09a	0.7 ± 0.33a	5.5 ± 0.44a	4.6 ± 0.58b
Yam	8.2 ± 0.12a	1.6 ± 0.19a	2.0 ± 0.05d	5.3 ± 0.12a	4.6 ± 0.58a

Each data represents the mean ± 2*σ* of the three replicates. Mean followed by the same letter in the same column are not significantly different (*p* > 0.05) from each other by the new Duncan’s multiple range test

## Discussion

The high survival rate of adult *A. fasciculatus* in cassava might be due to its high moisture, low antinutrient substance like tannin that could inhibit digestive enzymes, bind with proteins, and reduce absorption of food (Tawfiq et al. 2009) and only little mortality was recorded on it compared to the other food media. These properties of cassava promote its susceptibility to *A. fasciculatus*. However, the cocoyam flour recorded the highest beetle mortality after 10 days of the introduction of the insects this might be as a result of high saponin content. This view is in line with the report of Guo et al. (2018) and Chen et al. (2016) that described saponins as a natural substance with insecticidal property. Low saponins content could also be responsible for the low insects’ mortality on the cassava flour. Also, the highest number of eggs, larvae, pupae, and adults that emerged was recorded in the cassava flour while no larvae, pupae, and adults were found in the cocoyam flour. Tannin, alkaloid, phytate, and saponin are chemicals that have been noted by various authors to affect the feeding ability of insects thereby leading to their starvation. Yang et al. (2006) reported that tannin, saponin, alkaloids, phytate, and cardiac glycosides can inhibit the normal growth of insects. Therefore, the inability of the insects to develop in the cocoyam could be due to a higher concentration of these compounds.

Chijindu and Boateng (2008) reported a high number of eggs, larvae, and adult *A. fasciculatus* on fermented cassava chip for 14 days and this agreed with the results of this study. The highest weight loss was observed in cassava flour infested with the adult *A. fasciculatus* followed by the flour of plantain. Isah et al (2012) reported damage and weight loss to these staple foods by similar stored product pest. However, the cocoyam flour recorded no weight loss, and this was a result of no larvae, pupae, and adults that emerged from it. This could also be associated with the effect of the anti-nutritional factors that were present in this flour. These anti-nutritional factors must have affected the hatchability of the laid eggs or cause the inability of the immature to develop to adults due to the death of their larvae which cannot cast off their old exoskeleton which typically remained linked to the posterior part of the abdomen. Similarly, Chijindu and Boateng (2008) noted that cassava flour infested with adult *A. fasciculatus* experienced high weight loss after some period of storage. The proximate composition of food media can significantly affect the growth performance of A. fasciculatus, which can have implications for food security. A. fasciculatus is a pest insect that can cause significant damage to crops, particularly in tropical regions where it is prevalent. The insect feeds on a wide range of host plants, including cassava, yam, and plantain, which are important staple food crops in many African countries.

## Conclusions

The susceptibilities of these food products to *A. fasciculatus* decrease from cassava, plantain, yam to cocoyam. The susceptibility of these food media to *A. fasciculatus* infestations can depend on their moisture content. Foods with high moisture content, such as cassava and plantain, are more susceptible to insect damage than those with low moisture content, such as yam and cocoyam. Therefore, proper storage conditions, including drying and proper packaging, can help reduce the risk of *A. fasciculatus* infestations. There is a strong link between anti-nutritional compounds and growth performance or survival rate of *A. fasciculatus.* The susceptibility of processed and stored cassava, plantain, yam, and cocoyam to coffee bean weevil infestations depends on several factors, including their moisture content, nutrient composition, and antinutrient properties. Proper storage and handling practices, as well as selecting and managing food crops with specific nutrient profiles, are critical for developing effective pest management strategies and ensuring food security. Processed cocoyam should be prioritized for long term storage rather than for processed cassava in developing countries where there is no access to a good and improved post-harvest storage system for these staple foods. This study also provides informed knowledge required for the biotechnological improvement in these four staples food with desirable antinutrient content that can resist *A. fasciculatus.*

## Data Availability

The datasets used and/or analyzed during the current study are available from the corresponding author upon reasonable request**.**
